# A Razor’s Edge: Vascular Responses to Acute Inflammatory Lung Injury/Acute Respiratory Distress Syndrome

**DOI:** 10.1146/annurev-physiol-042222-030731

**Published:** 2024-02-12

**Authors:** David R. Price, Joe G.N. Garcia

**Affiliations:** 1 Department of Medicine, Division of Pulmonary and Critical Care Medicine, New York-Presbyterian Hospital/Weill Cornell Medical Center, New York, NY, USA; 2 Center for Inflammation Sciences and Systems Medicine, The Herbert Wertheim UF Scripps Institute for Biomedical Innovation & Technology, Jupiter, Florida, USA;

**Keywords:** ARDS, acute respiratory distress syndrome, vascular responses, endothelium, innate immunity, cell death, inflammation

## Abstract

Historically considered a metabolically inert cellular layer separating the blood from the underlying tissue, the endothelium is now recognized as a highly dynamic, metabolically active tissue that is critical to organ homeostasis. Under homeostatic conditions, lung endothelial cells (ECs) in healthy subjects are quiescent, promoting vasodilation, platelet disaggregation, and anti-inflammatory mechanisms. In contrast, lung ECs are essential contributors to the pathobiology of acute respiratory distress syndrome (ARDS), as the quiescent endothelium is rapidly and radically altered upon exposure to environmental stressors, infectious pathogens, or endogenous danger signals into an effective and formidable regulator of innate and adaptive immunity. These dramatic perturbations, produced in a tsunami of inflammatory cascade activation, result in paracellular gap formation between lung ECs, sustained lung edema, and multi-organ dysfunction that drives ARDS mortality. The astonishing plasticity of the lung endothelium in negotiating this inflammatory environment and efforts to therapeutically target the aberrant ARDS endothelium are examined in further detail in this review.

## 1. INTRODUCTION

Acute respiratory distress syndrome (ARDS) is a common, often fatal, inflammatory lung injury with an in-hospital mortality rate exceeding 40% (1). While the lung comprises numerous cell types that potentially contribute to the inflammation, immunity, and tissue fibrosis that determine the development and severity of ARDS, lung capillary endothelium, within a highly specialized location, is an essential contributor to ARDS outcomes. The endothelium has emerged as a spatially distributed network that interconnects the function/dysfunction of all organs in the human body, processes essential to both health and disease. This review addresses the direct participation of lung endothelial cells (ECs) in inflammatory/immune pathologies (*a*) via dynamic responses to extracellular environmental changes and participation in innate and adaptive immune responses by activating or suppressing immune cell function; (*b*) via expression of pattern recognition receptors (PRRs) and direct generation/secretion of key damage-associated molecular pattern proteins (DAMPs), which amplify the immune response; (*c*) via harboring genetic determinants of ARDS severity and outcomes; (*d*) as architects of ARDS resolution with resealing of the EC barrier to the pulmonary niche as inflammation, microbial invasion, and danger/damage signals abate; (*e*) and as a promising druggable target in ARDS.

The dramatic perturbation of lung EC monolayer barrier integrity, produced in the tsunami of inflammatory cascade activation, results in the formation of paracellular gaps between lung ECs first captured by electron microscopy studies by Bachofen & Weibel in 1977 (2). However, as ascertained from gene candidate studies, gene-targeted murine studies, and human biomarker analyses, it has been overwhelmingly clear that vascular mechanisms are directly linked to acute lung injury (ALI) and ARDS development and prognosis. A proverbial razor’s edge separates these adaptive responses from maladaptive disease-modifying mechanisms. In this review, we emphasize modes of experimental evidence that together provide compelling evidence that a vascular injury mechanism is maladaptive: (*a*) genetic polymorphisms in vascular-relevant genes that are mechanistically linked to protection from or risk of ALI/ARDS,(*b*) gene-targeting preclinical studies that recapitulate ALI/ARDS, and (*c*) vascular molecules causally linked to human ALI/ARDS development or prognosis.

Finally, this review focuses on the unmet need for effective therapeutics that target unchecked innate immunity-driven systemic hyperinflammation and unchecked vascular permeability in multi-organ dysfunction and ARDS mortality.

## 2. HOMEOSTATIC QUIESCENT AND ACTIVATED ENDOTHELIUM: ENDOTHELIAL CELLS AS IMMUNE EFFECTORS IN ACUTE RESPIRATORY DISTRESS SYNDROME

**Figure 1:**
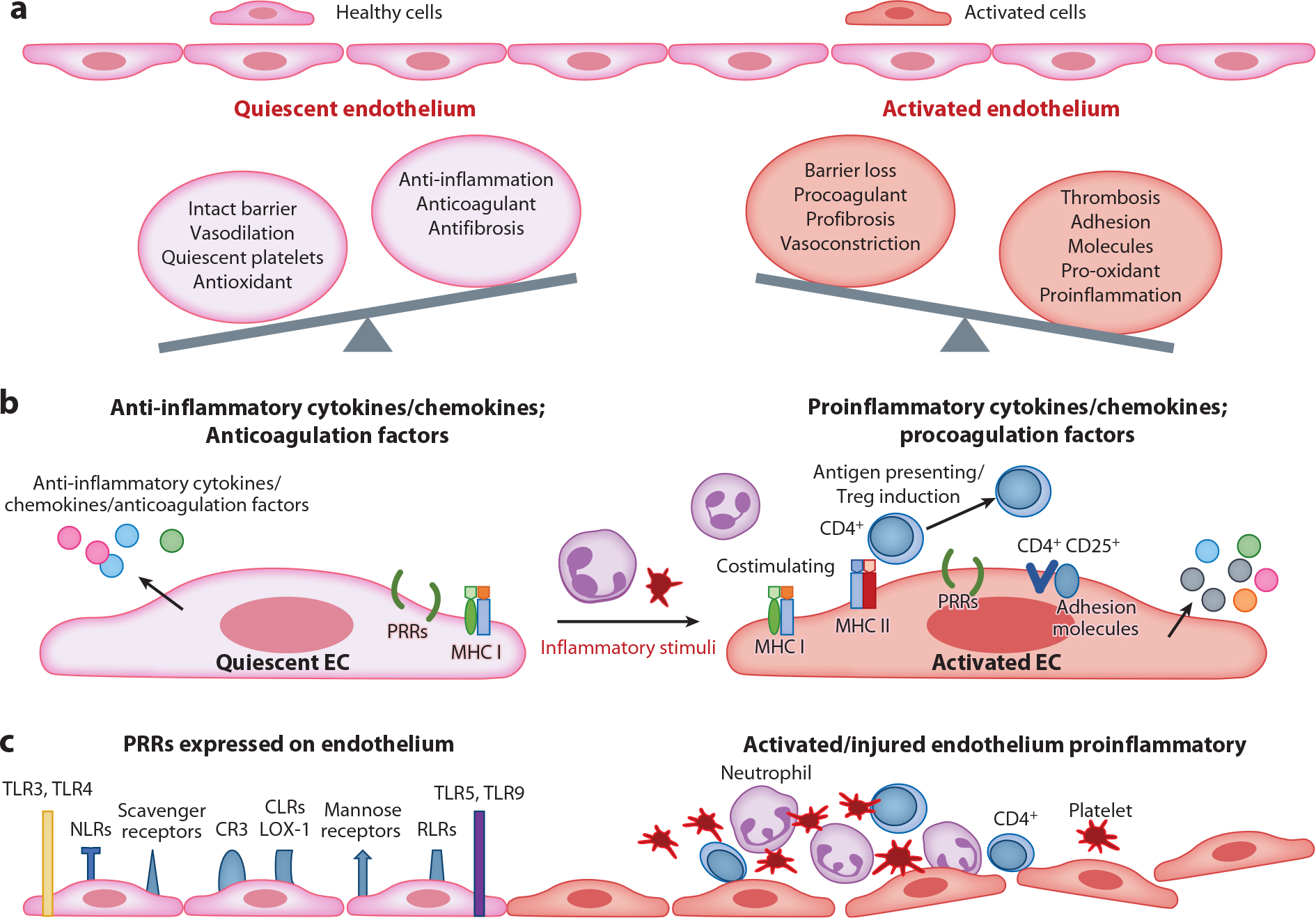
Homeostatic quiescent and activated endothelium. ECs as immune effectors in ARDS: (*a*) The quiescent lung endothelium is an intact monolayer of ECs defined phenotypically as exhibiting high barrier integrity and anticoagulant, antifibrotic, and anti-inflammatory properties. These homeostatic mechanisms are lost in ECs activated by proinflammatory agonists, PAMPs, and DAMPs. (*b*) Quiescent lung ECs constitutively release anti-inflammatory cytokines/chemokines and anticoagulation factors but may transform into nonprofessional immune effectors that are capable of presenting antigens (MHC I and II), de novo cytokine production, and costimulation of adaptive immune cells. (*c*) Lung ECs express multiple PRRs that allow for broad recognition of invading microorganisms and cell stress signals. Engagement of PRRs transforms quiescent ECs into activated proinflammatory ECs. Figure inspired by Reference 153. Abbreviations: ARDS, acute respiratory distress syndrome; CD, cluster of differentiation; CLR, C-type lectin receptor; CR3, complement receptor type 3; DAMP, damage-associated molecular pattern; EC, endothelial cell; LOX-1, lectin-like oxidized low-density lipoprotein receptor 1; MHC, major histocompatibility complex; NLR, nucleotide-binding oligomerization domain-like receptor; PAMP, pathogen-associated molecular pattern; PRR, pattern recognition receptor; RLR, retinoic acid-inducible gene-I-like receptor; TLR, Toll-like receptor; Treg, regulatory T cell.

Historically considered a metabolically inert cellular layer separating the blood from the underlying tissue, the endothelium is now recognized as a highly dynamic, metabolically active tissue that is critical to organ homeostasis, particularly the lung. Under homeostatic conditions, lung ECs in healthy subjects are quiescent, regulating vascular tone and coagulation/hemostasis, and exhibiting critical anticoagulant, antioxidant, and antifibrotic properties promoting vasodilation, platelet disaggregation, and anti-inflammatory mechanisms (Figure 1a). Importantly, the quiescent endothelium is a master regulator of lung fluid balance, forming an interactive intact monolayer barrier that ensures vascular integrity and appropriate permeability between blood and the lung interstitium to allow homeostatic diffusion of nutrients, oxygen, and metabolites to maintain autocrine and paracrine function. Lung ECs are interconnected but exhibit remarkable diversity in structure and function along the vascular axis to achieve their site-specific mandates, with capillary endothelium serving as gatekeepers of gas exchange, fluid balance, and leukocyte diapedesis.

The quiescent EC phenotype is rapidly and radically altered upon exposure to environmental stressors, infectious pathogens, or endogenous danger signals, each of which can transform lung ECs into an effective and formidable regulator of innate and adaptive immunity. As a critical function of innate immune cells is to monitor the blood for invading pathogens, lung ECs serve as frontline defenders against pathogens and are often the first cell to encounter bloodborne danger signals. Activated ECs are central to innate immunity responses, choreographing complex molecular and ultrastructural changes that allow innate immunity responders access to the pulmonary niche while simultaneously upregulating homeostatic programs to minimize inflammatory damage to the lung parenchyma.

Activated lung ECs regulate evolutionarily conserved innate immunity and inflammatory cascades by exhibiting features common to professional innate immune effector cells. A poorly appreciated phenotypic feature shared by lung ECs with immune effectors is the apparent capacity to exhibit a phagocytic function with physical removal/phagocytosis of pathogens and danger signals. Although considered nonprofessional phagocytes compared to neutrophils, monocytes, and macrophages, ECs have the capacity to phagocytose, internalize, and remove bacteria, fungi, and inorganic particulates such as asbestos fibers, as well as circulating apoptotic ECs (3–6).

A second key feature shared by lung ECs with immune effectors is the capacity for cellular migration. EC cellular migration is critically involved in angiogenesis and wound healing and is driven by chemotactic cues, haplotaxis (directional cell movement by the extracellular matrix), or environmental mechanotactic cues, including the mechanical stress of the mechanical ventilator. Lung EC migration is critical to restoring vessel integrity in damaged vessels involving spatially directed activation of the EC cytoskeleton, which drives closure of inflammation-induced paracellular gaps between lung ECs (7).

Lung ECs also serve as nonprofessional antigen-presenting cells, a critical phenotypic feature of immune effectors, by processing and presenting EC antigens to T cells (8) (Figure 1b). ECs express major histocompatibility complex (MHC) I and II and costimulatory molecules to selectively regulate the influx of antigen-specific cells to sites of injury, processes critical to an effective immune response. Among lung ECs, capillary ECs exhibit the highest MHC I protein expression, demonstrating heightened EC immune surveillance at the nexus of the vascular compartment and the lung niche (9). These leukocyte–EC interactions directly influence T cell function, adaptive immunity expression of surface receptors for pathogen and danger/damage signals, and cytokine production by both ECs and immune cells (Figure 1b).

**Figure 2:**
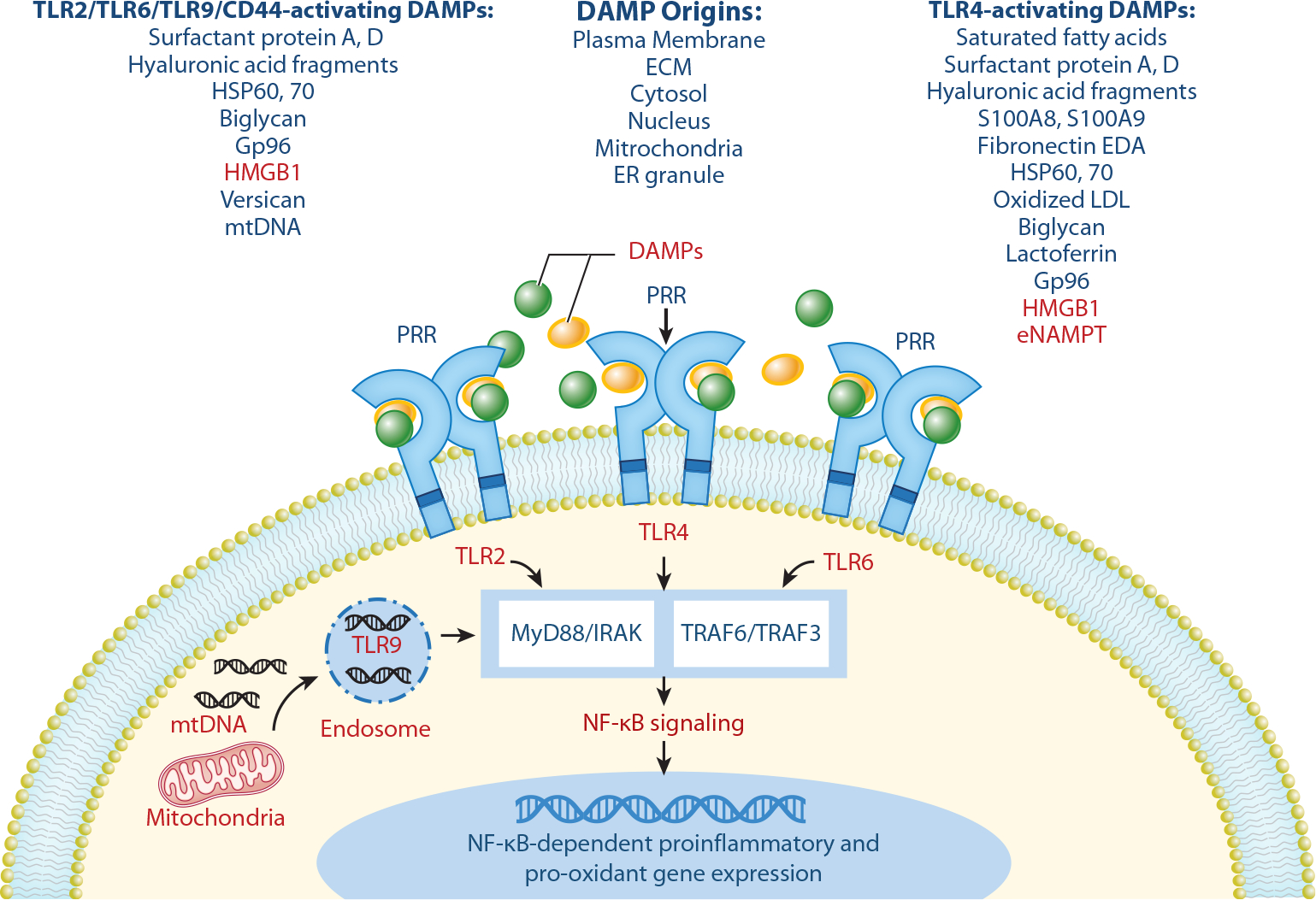
Endogenous alarm signals or DAMPs engage endothelial TLRs to initiate or amplify the innate immunity inflammatory response. Lung ECs express diverse TLRs and generate diverse DAMPs that participate in innate immunity responses, often as the first responder. Both surface TLRs (TLR2, TLR4, TLR6) and endosomal TLR9 allow ECs to serve as early sentinels to metabolic disruption and increasing cytokine production essential to the pathobiology of ALI/ARDS. Red font denotes molecules and signaling pathways highlighted in this review. Abbreviations: ALI, acute lung injury; ARDS, acute respiratory distress syndrome; CD, cluster of differentiation; DAMP, damage-associated molecular pattern; EC, endothelial cell; ECM, extracellular matrix; EDA, extra domain A; eNAMPT, extracellular nicotinamide phosphoribosyltransferase; ER, endoplasmic reticulum; Gp96, glycoprotein 96; HMGB1, high-mobility group box 1; HSP, heat shock protein; IRAK, interleukin-1 receptor-associated kinase; LDL, low-density lipoprotein; mtDNA, mitochondrial DNA; MyD88, myeloid differentiation primary response protein 88; NF-κB, nuclear factor kappa B; PRR, pattern recognition receptor; S100A8, S100 calcium-binding protein A8; S100A9, S100 calcium-binding protein 9; TLR, Toll-like receptor; TRAF, tumor necrosis factor receptor–associated factor.

Similar to professional immune effectors, lung ECs express multiple PRRs on the EC surface (Figure 1c), providing a key mechanism for lung ECs to serve as danger sensors. Lung ECs are early sentinels to detect pathogens in the circulation and elicit critical innate immune danger signal amplification via PRR engagement with pathogen-associated molecular patterns (PAMPs) on the invading pathogen surface [lipopolysaccharide (LPS) is the classic PAMP] or with DAMPs, which are endogenous danger signals released upon cellular stress or tissue injury (e.g., necroptotic/pyroptotic or damaged cells; see Section 3). PRR ligation induces innate immunity-mediated inflammation, with ECs expressing an impressive array of PRRs, including Toll-like receptors at the cell surface (TLR1, TLR2, TLR4, TLR5, TLR6, TLR11) that detect extracellular PAMPs and DAMPS, and TLRs localized to intracellular vesicles such as endosomes, lysosomes, or vesicles (TLR3, TLR7, TLR8, TLR9), which detect intracellular PAMPs’ viral and bacterial DNA (8, 10–12). ECs predominately express TLR4, the central PRR in EC-elicited innate immunity inflammation and a highly promiscuous receptor activated by bacterial and viral PAMPs and a wide array of DAMPs (Figure 2). TLR2, the receptor for gram-positive and fungal wall components, is expressed at low levels in ECs; however, both TLR2 and TLR4 are prominently upregulated in ECs exposed to cytokines, PAMPs, and DAMPs (13). ECs also express important downstream adaptor molecules for TLR signaling, including myeloid differentiation factor-2 (MD-2) and myeloid differentiation primary response protein 88 (MyD88) (5). While MyD88 is a critical intracellular adaptor molecule in the canonical TLR signaling cascade, MD-2 is associated with the extracellular domain of TLR4 required for LPS signaling cascade involving MyD88,tumor necrosis factor receptor–associated factor (TRAF)3/6, interleukin-1 receptor-associated kinase (IRAK), nuclear factor kappa B (NF-κB), and mitogen-activated protein kinase (MAPK) family kinases (8, 14) (Figure 2).

Other EC surface-expressed PRRs include C-type lectin receptors (including mannose 1 receptor), nucleotide-binding oligomerization domain (NOD)-like receptors (NLRs), retinoic acid-inducible gene-I-like receptors (RLRs), and scavenger receptors including lectin-like oxidized low-density lipoprotein receptor 1 (LOX-1), CD36, and RAGE receptors (8, 15–18) (Figure 1c). While lung EC TLRs monitor the extracellular environment, cytosolic NOD proteins provide intracellular recognition of microbes. NLRs, including NOD1 and NOD2, are upregulated in the lung ECs in response to bacteria (19) and viruses (20), amplify EC responses through NF-κB pathways, and are essential components of a cytoplasmic surveillance pathway in lung ECs.

Along with PRR expression, lung ECs retain the capacity to amplify the innate immune inflammatory response by the production and release of cytokines, chemokines, DAMPs, and PAMPs, which bind cell receptors/PRRs on innate immunity cells, including ECs, to initiate or amplify the innate inflammatory response. De novo EC cytokine production is tightly regulated, with multiple proinflammatory signaling pathways converging to increase NF-κB-driven EC production/secretion of inflammatory cytokines [interleukin (IL)-6,IL-8,monocyte chemoattractant protein-1 (MCP-1), tumor necrosis factor alpha (TNF-α), IL-1α, IL-1β]. The number of DAMPs identified continues to increase (21–23), with EC-derived DAMPs serving as important danger sentinels (Figure 2). DAMPs promote immune responses via ligation of PRRs, producing both infection-induced inflammation and sterile inflammation, exemplified by the profound noninfectious inflammatory lung injury produced by exposure to mechanical ventilation [ventilator-induced lung injury (VILI) (24)], a significant contributor to ARDS mortality (25).

Two interesting DAMPs secreted from multiple tissues, including lung ECs, are the high-mobility group box 1 (HMGB1) and extracellular nicotinamide phosphoribosyltransferase (eNAMPT), whose expression is increased by exposure to hypoxia, cellular stress, and increased mechanical stress (26–28), with eNAMPT plasma levels elevated by bacterial/viral infection, sepsis, hypoxia, ischemia/reperfusion, radiation, and trauma (29–31). Both proteins exhibit key homeostatic intracellular functions but profound proinflammatory properties when secreted. Secretion of either HMGB1, an intracellular nonhistone nucleoprotein, or eNAMPT, an intracellular enzyme involved in nicotinamide adenine dinucleotide (NAD) biosynthesis (24, 32), results in NF-κB pathway-driven proinflammatory gene expression. Secreted HMGB1 binds to specific PRRs (TLR2, TLR4, TLR9, RAGE) (33–35) to release inflammatory factors involved in sepsis-induced pyroptosis pathways. The TLR4-binding DAMP, eNAMPT, also elicits profound NF-κB-driven inflammatory lung injury, processes involved in ARDS/VILI pathobiology (24, 32, 36). As shown in Figure 2, other PRR-binding, EC-derived DAMPs, such as S100 proteins and heat-shock proteins, are released extracellularly as lytic cell death products (see Section 3). These lytic cell death products produce robust NF-κB-dependent activation of inflammatory and prooxidant gene transcription, including genes related to leukocyte extravasation, and drive further expression of PRRs and DAMPs, including eNAMPT and HMGB1 (37).

Finally, in the transition from quiescent ECs to activated immune effectors, activated lung ECs upregulate adhesion molecules, such as vascular cell adhesion molecule (VCAM) and intercellular adhesion molecule (ICAM), to enhance immune/EC interactions by physically linking ECs to platelets and leukocytes, resulting in mutual cell activation and de novo expression of cytokines and adhesion molecules that amplify local inflammation. Lung ECs release chemotactic factors creating chemotactic gradients for immune cells and facilitating platelet/polymorphonuclear leukocyte (PMN) aggregation, leukocyte extravasation, and increased expression/secretion of P-selectin (38), PSGL1, a P- and E-selectin glycoprotein ligand, and IL-8 (39). Activated ECs mobilize P-selectin from Weibel-Palade bodies to the cell surface to facilitate the influx of leukocytes and regulatory T cells (Tregs) to sites of injury via PMN rolling, diapedesis, and platelet/PMN aggregation as the activated capillary endothelium simultaneously awakens to its essential function of increasing lung permeability during invoked inflammation.

## 3. MECHANISMS OF VASCULAR MALADAPTATION AND INCREASED PERMEABILITY IN ACUTE LUNG INJURY/ACUTE RESPIRATORY DISTRESS SYNDROME

### 3.1. Vascular Cytoskeletal Protein Involvement in Maladaptive Permeability

**Figure 3:**
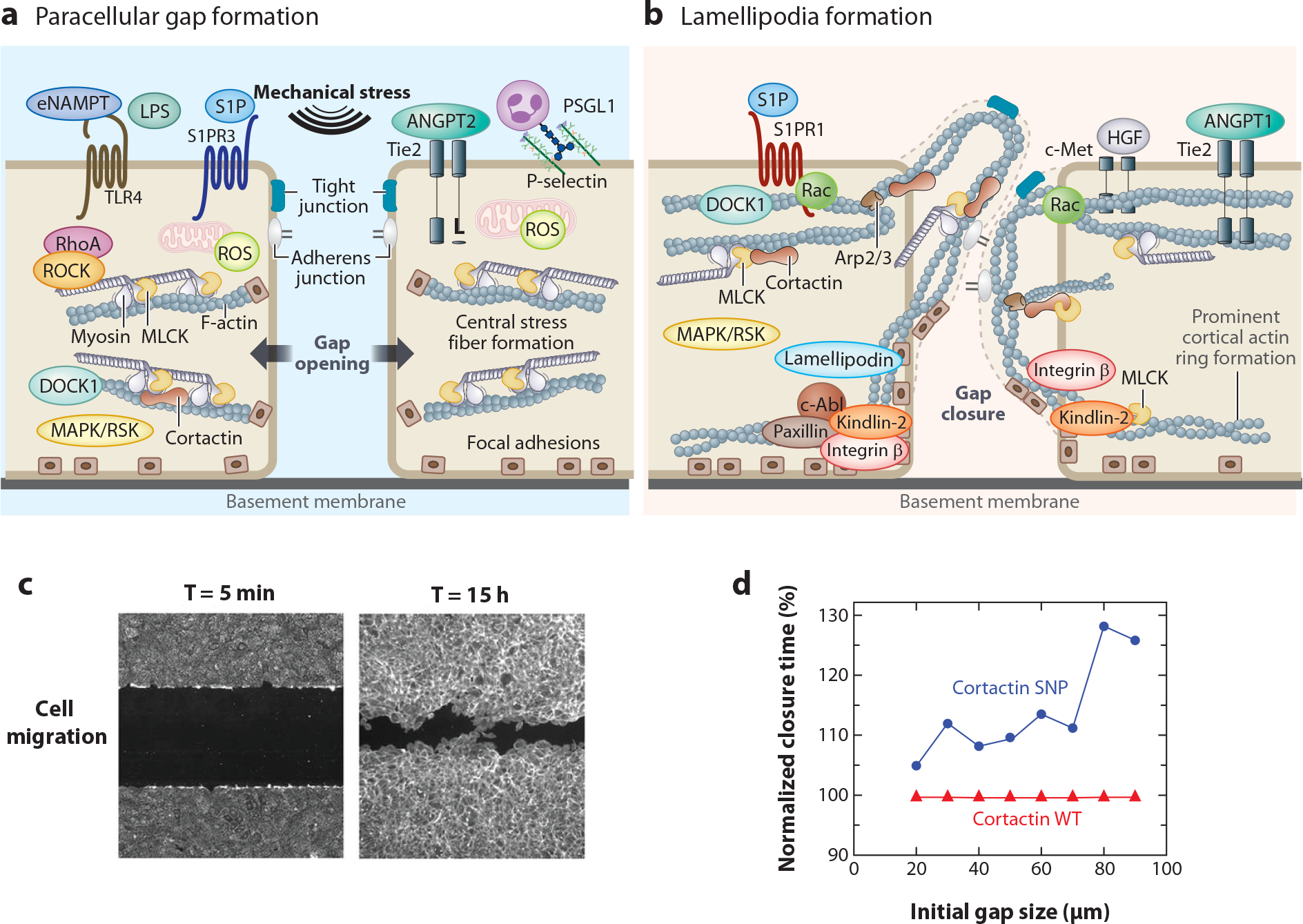
The opposing mechanisms of paracellular gap formation and lamellipodia formation in ALI/ARDS. (*a*) Multiple permeability pathways converge on RhoA GTPase/ROCK-mediated activation of nmMLCK, resulting in elevated MLC levels and actin filament contraction. Signaling pathways elicited by ligation of surface receptors such as TLR4, S1PR3, and Tie2 result in EC paracellular gap formation and profound vascular leak. (*b*) Inflammatory agonist-driven increases in EC permeability are countered by barrier-enhancing receptors ligated by platelet-derived ANGPT1 (Tie2), S1P (S1PR1), and HGF (c-Met) to resolve EC permeability. These signaling pathways involve the essential participation of Rac1 regulators (DOCK1), cytoskeletal effectors (nmMLCK, cortactin), and focal adhesion effectors (integrin β, kindlin-2) to increase cortical actin formation and lamellipodial protrusions to facilitate gap resolution. (*c*) Using a microfabricated platform, ECs demonstrate normal cellular migration time from 5 min to 15 h. (*d*) Cortactin plays an important role in EC barrier restoration. ECs with the cortactin SNP S484N grown in monolayer around removable circular stencils to create EC gaps have delayed closure time compared with WT ECs. Delayed closure time with the cortactin SNP is linked to a larger initial gap size. Panel *d* adapted from Reference 109. Abbreviations: ALI, acute lung injury; ANGPT1, angiopoietin 1; ARDS, acute respiratory distress syndrome; Arp, actin-related protein; c-Abl, cellular Abelson kinase; c-Met, cellular hepatocyte growth factor receptor; DOCK1, dedicator of cytokinesis 1; EC, endothelial cell; eNAMPT, extracellular nicotinamide phosphoribosyltransferase; F-actin, filamentous actin; GTPase, guanosine triphosphatase; HGF, hepatocyte growth factor; LPS, lipopolysaccharide; MAPK, mitogen-activated protein kinase; MLCK, myosin light chain kinase; nmMLCK, nonmuscle MLCK; P-selectin, platelet selectin; PSGL1, P-selectin glycoprotein ligand 1; Rac, Ras-related C3 botulinum toxin substrate; RhoA, Ras homolog family member A; ROCK, Rho-associated protein kinase; ROS, reactive oxygen species; RSK, ribosomal S6 kinase; S1P, sphingosine-1-phosphate; S1PR3, S1P receptor 3; SNP, single nucleotide polymorphism; T, time; TLR, Toll-like receptor; WT, wild-type.

Unremitting inflammation, coupled to the persistent loss of vascular barrier integrity, sustained lung edema, and multi-organ dysfunction, is a major driver of ARDS mortality. Lung ECs are of the continuous subtype, with intercellular junctions and anchored to a continuous basement membrane. This continuous subtype feature is particularly true for lung capillary ECs, which exhibit a highly restrictive barrier (40), a critical factor retarding the development of alveolar flooding. Current concepts of lung vascular barrier regulation involve highly dynamic lung EC barrier regulation by the actomyosin EC cytoskeleton, which choreographs spatially directed increases in cellular tension. This EC barrier regulation can either favor barrier-disruptive contractile forces, thus promoting vascular permeability, or, conversely, favor barrier-protective tethering forces, driven by cytoskeletal proteins, which promote barrier stabilization and restoration of the intact EC barrier during recovery (41, 42) (Figure 3).

Although there are >100 known actin-binding proteins, two critical lung cytoskeletal effector proteins, encoded by recognized ARDS candidate genes, are central to lung EC barrier regulation: the multifunctional Ca^2^+/calmodulin-dependent nonmuscle myosin light chain kinase isoform (nmMLCK) and its cytoskeletal-binding partner, cortactin. These EC actin-binding cytoskeletal effectors are involved in (*a*) regulation of vascular barrier processes, including inflammatory leukocyte trafficking (43, 44); (*b*) vascular responses to ventilator-generated mechanical stress (45); and (*c*) harboring of genetic variants that are overrepresented in Blacks with ARDS and contributing to ARDS susceptibility and mortality (46, 47). nmMLCK exhibits dual functionality as a proinflammatory effector contributing to lung EC reactive oxygen species (ROS) formation (48) and barrier disruption (49), but also a barrier-protective molecule in response to specific environmental cues. Both nmMLCK and cortactin are intimately involved in maintaining barrier integrity in quiescent ECs and are essential to the formation of a cortical actomyosin band at the cell periphery. This cortical actomyosin band promotes monolayer integrity via intercellular adherens junctions linked to the actin cytoskeleton by catenins that determine restrictive cell-to-cell junctional integrity and by cell-to-matrix tethering forces (42, 50).

Because of its enormous surface area, the lung vasculature is particularly sensitive to excessive generation of ROS and the activation of innate immunity inflammatory cascades. Inflammation-mediated activation of the cytoskeletal contractile apparatus in ARDS, involving proinflammatory mediators [thrombin, histamine, vascular endothelial growth factor (VEGF)], and PRR ligation by PAMPs (such as LPS) and DAMPs (such as eNAMPT), induces massive disruption of the alveolar-capillary barrier, resulting in alveolar flooding. The TLR4 signaling cascade that increases EC permeability involves dysregulated activity of a mechanosensitive ion channel protein, Piezo1, and NOX4, a key ROS-producing target implicated in the pathogenesis of both sepsis and ARDS (51, 52). Inflammatory signaling promotes the dissolution of the EC cortical actin band, with formation of cytoplasmic actin stress fibers that generate tensile forces to cause disruption of cell–cell and cell–matrix contacts and the loss of monolayer integrity with formation of paracellular gaps (41) (Figure 3a). A driving force behind the activation of the cytoskeletal contractile apparatus is nmMLCK, which phosphorylates myosin light chains to drive actin-myosin contraction (53) and regulate EC barrier function (53, 54), angiogenesis (55), EC apoptosis (56), leukocytic trafficking (44), and vascular responses to mechanical stress (57, 58).

Proinflammatory agonists, cytokines, DAMPs, PAMPS, and increased mechanical stress all significantly increase nmMLCK protein expression via hypoxia-inducible factors (HIF-1a/HIF-2a) (58) with unique negative *nmMYLK* transcriptional regulation by nuclear factor erythroid 2-related factor 2 (NRF2)/antioxidant elements (59). These proinflammatory mediators robustly increase nmMLCK activity to elicit spatially localized central contraction, paracellular gap formation, and disruption of the lung vascular barrier in vitro and in vivo (45, 49). The Ras homolog family member A (RhoA) guanosine triphosphatase (GTPase) directly binds and activates nmMLCK and is a significant promoter of EC permeability (60, 61). RhoA/Rho kinase phosphorylation/inactivation of the myosin light chain phosphatase elevates MLC levels, inducing EC contraction and vascular leak (62, 63).

Following established inflammation and vascular leak, key EC homeostatic mechanisms are evoked to restore the impaired EC barrier, a critical step in survival from ARDS. The cellular movements of nmMLCK and cortactin are essentially involved in barrier-restoring actin assembly/disassembly to reform the strong cortical actin band and drive lamellipodial protrusions to promote paracellular gap closure and restore EC barrier integrity (64) (Figure 3b,c). These findings underscore the complexity and duality of nmMLCK’s role in vascular barrier regulation and dynamic phasic barrier regulation (inflammation development versus inflammation resolution). Spontaneous lung EC barrier recovery is accelerated by endogenous EC barrier enhancers, such as sphingosine-1-phosphate (S1P), angiopoietin 1 (ANGPT1), and hepatocyte growth factor (HGF) (50, 65, 66). Another critical participant in the resolution phase of ARDS-induced vascular permeability is the Rac1 GTPase (51, 67), which stabilizes the EC barrier to decrease stress fiber formation and barrier dysfunction. The DOCK1-ELMO1 complex is a critical regulator of Rac GTPases and lung EC barrier integrity and participates in the formation of lamellipodia containing nmMLCK, cortactin, focal adhesion components, and lamellipodin to facilitate paracellular gap closure and enhance vascular integrity (51) (Figure 3b). Activation of the TLR4 signaling cascade markedly reduces DOCK1 and ELMO1 expression, influencing the severity of inflammatory lung injury in ARDS/VILI models (51).

### 3.2. The Disrupted Sphingosine-1-Phosphate Axis

In the quiescent endothelium, the bioactive lipid mediator S1P is an endogenous EC barrier-enhancing, cytoskeletal protein–remodeling agonist that serves as a counterweight to inflammation-induced barrier destabilizers associated with pathogenic leak. Erythrocytes and ECs maintain high plasma concentrations of S1P, the product of sphingosine kinase (SphK)-induced phosphorylation of sphingosine, representing a constitutive well of protective substrate for the quiescent endothelium (68, 69).

Proinflammatory cytokines, growth factors [e.g., platelet-derived growth factor (PDGF), VEGF], oxidative stress, hypoxia, sheer stress, and cyclic stretch significantly increase SphK expression, representing an adaptive effort to maintain S1P levels when facing persistent pathogenic vascular leak. Activated platelets provide an additional reserve of S1P (70) to counter disruptive barrier forces. The development of pathogenic lung edema in ARDS suggests these adaptive mechanisms eventually fail. Serum S1P levels decrease in ARDS patients and are associated with worsened clinical outcomes (44). Global or plasma deletion of *Sphk1* in mice increases vascular leak and worsens murine survival (68, 69, 71–73). Additional mechanisms compromising S1P activity in ALI/ARDS include the loss of S1P carrier proteins, leading to lower S1P plasma levels (74).

S1P exerts its quiescent, potent EC barrier-regulatory function by ligating the sphingosine1-phosphate receptor-1 (S1PR1). S1PR1 is highly expressed on lung ECs where S1P/S1PR1-mediated EC barrier enhancement is Rac1 dependent and involves G_i_-PI3K-Tiam1 signaling to strengthen the vascular barrier through adherens and tight junction assembly, cytoskeletal reorganization, and the formation of focal adhesions (75) (Figure 3b). In the murine lung, inhibition of S1PR1 is associated with vascular leak (76), representing a pathogenic mechanism of lung edema formation in ARDS.

Alternatively, S1P disrupts barrier integrity in the inflamed lung through sphingosine-1-phosphate receptor-3 (S1PR3), the receptor encoded by the ARDS candidate gene *S1P3*, which in contrast to S1PR1-mediated Rac1-signaling, exerts permeability in a RhoA-dependent manner. Proinflammatory stimuli upregulate EC S1PR3, and increased plasma S1PR3 levels are associated with hyperpermeability in mice and ARDS mortality (77). In addition, genetic variants overexpressed in non-Hispanic Whites decrease S1PR3 promoter activity and lower plasma S1PR3 levels, offering protection against ARDS development (78).

### 3.3. The Disrupted Angiopoietin Axis

In addition to S1P, the quiescent endothelium enjoys additional barrier enhancement from ANGPT1 and constitutive signaling from Tie2, the ANGPT tyrosine kinase receptor. This homeostatic interaction between tonically expressed ANGPT1 and the receptor Tie2 maintains Tie2 phosphorylation, enhancing barrier integrity by activating Rac1 (79, 80). Constitutive ANGPT1 signaling also regulates the expression of the barrier-disruptive angiopoietin 2 (ANGPT2) thru Akt phosphorylation of forkhead box O1 (FOXO1) transcription factor, repressing ANGPT2 transcription (81). Ironically, in the absence of inflammatory conditions, ANGPT2 assumes a barrier-promoting function, defying its role as a quintessential barrier disruptor to contribute to EC barrier enhancement via Tie2 agonism (82). However, proinflammatory cytokines promote previously quiescent ECs to release ANGPT2 from EC Weibel-Palade bodies, disrupting the EC barrier. This ANGPT2-mediated barrier disruption is opposed by ANGPT1 and PDGF released from activated platelets (83).

However, sustained induction of circulating ANGPT2 overwhelms these adaptivemechanisms, as demonstrated in ANGPT2-challenged mice that develop pulmonary leak (84). ANGPT2 additionally augments a switch of vascular responsiveness by sensitizing EC toward TNF-α and modulating TNF-α-induced expression of adhesion molecules, including ICAM-1 and VCAM-1 (85), driving an accelerated feedback loop increasing de novo ANGPT2 expression and hyperpermeability. Thus, ANGPT2-elicited increases in lung permeability involve multiple mechanisms, including RhoA-dependent increases in nmMLCK-driven activation of the EC contractile apparatus (84). ANGPT2 also promotes the loss of Tie2 signaling, a particularly maladaptive vascular response that induces robust Rho-kinase/MLCK-mediated MLC phosphorylation, EC contraction, gap formation, and barrier disruption (86). Tie2 disruption also cross talks to maladaptive coagulopathy and precedes overt activation and consumption of platelets and fibrin accumulation (87). These data place Tie2 and the angiopoietin axis at the nexus of injurious vascular responses in ALI/ARDS pathogenesis.

### 3.4. Maladaptive Platelet Responses and Coagulopathy in Acute Lung Injury/Acute Respiratory Distress Syndrome

Platelets are activated in early ALI by a variety of factors, including inflammatory cytokines (e.g., TNF-α), products of coagulation (e.g., thrombin), and platelet-activating factor, a bioactive phospholipid released from a variety of immune cells and ECs. Activated platelets release inflammatory cytokines such as CD40 ligand (CD40L) and ROS, which directly activate ECs to secrete chemokines, including IL-8 and MCP-1, and to express adhesion molecules, including E-selectin, VCAM-1, and ICAM-1, thereby generating signals for the recruitment and extravasation of leukocytes to injury sites (88).

Activated platelets also play a critical role in recruiting neutrophils to the lung via direct cellular interactions involving GPIIbIIIa/Mac-1 and P-selectin/PSGL1 or neutrophil activation through platelet secretory products, such as P-selectin stored in alpha granules of platelets and Weibel-Palade granules of ECs. Neutrophil-platelet interactions promoted mutual cell activation and secondary capture of neutrophils and other leukocytes, resulting in endothelial injury (89). Platelet depletion diminishes neutrophil accumulation in the lung’s intravascular, interstitial, and alveolar spaces, demonstrating platelets’ central role in neutrophil accumulation in ALI.

Platelets are also essential to neutrophil extracellular trap (NET) formation, a web-like structure composed of DNA, histones, and antimicrobial proteins, and a key activator of the pulmonary endothelium in ALI/ARDS pathogenesis. NETs are part of the innate immune response and play a role in host defense but can also contribute to tissue damage and inflammation. NETs are present in the lungs and plasma of humans with transfusion-related acute lung injury (TRALI) and in the plasma of ALI patients. In experimental TRALI models, targeting platelet activation or NET components decreases NET formation and lung injury (90).

Platelets also enhance the integrity of the microcirculation (91), with thrombocytopenia increasing capillary permeability and accelerating fluid and protein extravasation (92). This symbiosis between activated platelets and pulmonary EC health is reflected in ALI/ARDS plasma. ARDS blood vascular proteomics show that low platelet levels track with loss of platelet-derived trophogens, including CD40 ligand (CD40LG), glycoprotein 6 (GP6), ANGPT1, matrix metalloproteinase 9 (MMP9), PDGFA, and PDGFB (93). ANGPT2 levels are highest in individuals with low platelet numbers who exhibit the highest mortality, pointing to the maladaptive effect of platelet depletion on sustained vascular barrier instability and identifying platelet levels as a barometer of vascular health in ALI/ARDS (87, 93). The platelet/endothelial association is agnostic of the etiology of platelet depletion: Both low platelets from consumptive disseminated intravascular coagulation (87) and malignancy-related (93) low platelets link to high ANGPT2 in ALI/ARDS-inducing diseases.

### 3.5. Maladaptive Vascular Cell Death

Abnormally high levels of inflammatory cytokines, viral infections, and escalating intracellular stress signals can induce injured ECs to upregulate cell death programs, resulting in regulated cell death, barrier disruption, and vascular leakage into surrounding tissues (94). While the death/loss of a lung EC invokes the idea of endothelial permeability through physical barrier loss, this dropout hypothesis is not supported by electron microscopy studies of ARDS lungs, showing either intact endothelial monolayer ultrastructure (2) or EC vacuolization (95) in contrast to profound, widespread interstitial edema. Instead, a growing body of preclinical and translational data identified vascular cell death as functionally linked to ALI/ARDS lung injury.

Regulated cell death can be further categorized by the eventual fate of cell membrane integrity, with implications for the inflammatory potential of the dying cell. Apoptosis, the archetypal form of regulated cell death, executes cell death without the loss of plasma membrane integrity, minimizing the leak of intracellular DAMPs and representing an immunologically quiet form of cell death. In contrast, necroptosis and pyroptosis are lytic cell death programs characterized by organelle swelling, plasma membrane rupture, and leakage of a torrent of intracellular DAMPs, including mitochondrial DNA (mtDNA) or HMGB1 (see Figure 2), to drive immune responses. This review focuses on the evolving role of lytic endothelial cell death programs, including necroptosis and pyroptosis, in lung injury development.

The necroptosis pathway is regulated by receptor-interacting protein kinases 1 and 3 (RIPK1 and RIPK3) and the downstream executioner, pseudokinase mixed-lineage kinase domain–like (MLKL) (96). Under conditions of caspase 8 depletion or cellular inhibitor of apoptosis protein deficiency,RIPK3-mediated phosphorylation of MLKL within the necrosome is the terminal step in necroptosis execution, leading to cell membrane lysis and the release of cellular contents, including intracellular DAMPs.

Endothelial necroptosis is causative of lung permeability, systemic inflammation, and coagulopathy in TNF-induced shock (97). This effect is independent of neutrophil recruitment, suggesting that the necroptotic EC can regulate permeability independent of neutrophils. EC necroptosis is activated by diverse ARDS stimuli, including allogenic red blood cells (98), hemin (99), heat stress (100), and endotoxin (through TLR4) (101), eliciting EC barrier dysfunction, in part through VE-cadherin disassembly and actin cytoskeleton remodeling (101). Notably, the vascular destabilizing factor ANGPT2 induces lung epithelial necroptosis and pulmonary edema, establishing a paracrine mechanism of necrotic cell death induction in EC barrier regulation (102). Whether ANGPT2 induces endothelial RIPK3-mediated necroptosis in barrier regulation remains unanswered, but this has been observed in human ARDS lungs and blood proteomics analyses (93).

Pyroptosis is an additional form of lytic cell death that requires the inflammasome signaling platform to produce caspases capable of activating gasdermin proteins (specifically GSDMD and GSDME). In mice, caspase 11 cleavage of GSDMD induces the release of the active membrane pore-forming GSDMD peptide, leading to cellular swelling and membrane rupture. In macrophages and ECs, cytoplasmic LPS delivered by microvesicles induces intracellular LPS-sensing pathways, leading to caspase 11 (or caspase 1/4/5 in humans)-mediated pyroptotic cell death, mimicking a host defense against gram-negative bacterial infections. Microvesicle-packaged LPS induces endothelial pyroptosis in mediating murine endotoxemic-induced lung injury (103).

## 4. HETEROGENEITY OF VASCULAR RESPONSE TO ACUTE LUNG INJURY/ACUTE RESPIRATORY DISTRESS SYNDROME AND THE PULMONARY NICHE

### 4.1. Genetic Influences on Maladaptive Mechanisms that Increase Susceptibility to Vascular Injury in Acute Respiratory Distress Syndrome

The COVID-19 pandemic highlighted the existence of severe ARDS racial disparities, with disproportional mortality in Blacks and Latinos, confirming increased susceptibility and disproportionate adjusted mortality in sepsis and ARDS (104, 105). A genetic basis for ARDS disparities has been suggested (106, 107), with single nucleotide polymorphisms (SNPs) identified in vascular inflammation- and permeability-regulating genes, thereby supporting ECs’ critical role in ARDS pathophysiology (Table 1). For example, promoter SNPs alter the transcriptional activity of *MYLK*, the gene encoding nmMLCK (see Section 3), induced by proinflammatory stimuli (LPS, TNF-α, ventilator mechanical stress) via HIF activities (58). In addition, *MYLK*-coding SNPs are overrepresented in Blacks (46, 108), conferring increased risk and severity of sepsis/trauma-induced ARDS.A coding SNP in *CTTN* (47),the gene encoding cortactin, is similarly significantly overrepresented in Blacks with ARDS and increases the severity of sepsis, sickle cell disease, and ARDS (47). Functionally, these *MYLK*- and *CTTN*-coding SNPs delay lung EC barrier recovery (47, 64, 109–111), with Figure 3d depicting the dramatic delay in gap closure in EC harboring the cortactin coding SNP (109). *MYLK*- and *CTTN*-coding SNPs also increase the risk of severe asthma in individuals of African descent (112). Finally, increased race-specific epigenetic *MYLK* regulation is observed in ARDS (113).

Similar to *MYLK*, expression of *NAMPT*, the gene encoding the novel DAMP and TLR4 ligand, eNAMPT (see Section 2), is also induced by ARDS stimuli, with *NAMPT* promoter SNPs significantly altering promoter activity and increasing levels of circulating eNAMPT (114–117). *NAMPT* SNPs confer increased susceptibility and severity of ARDS (reduced ventilator-free days, increased ARDS mortality) in Blacks and non-Hispanic Whites (32, 118).

The selectins (E-selectin, L-selectin, P-selectin) are a versatile family of transmembrane glycoproteins that mediate leukocyte tethering and rolling interactions with activated ECs, an early and essential part of the innate immune inflammatory response. PSGL1 is an extensively characterized selectin ligand encoded by *SELPLG,* a highly novel ARDS susceptibility gene among Blacks and non-Hispanic Whites. Genome-wide association studies (GWAS) in Blacks with ARDS identified a coding SNP that influences the activity of the *SELPLG*-encoded PSGL1 (119). Similar to *MYLK*, *CTTN*, and *NAMPT*, ARDS stimuli increase *SELPLG* promoter activity via the participation of transcription factors that include HIF-1α/HIF-2α and NRF2 (120).

As detailed in Section 3, S1PR1 and S1PR3 are major barrier-regulatory G protein–coupled receptors highly expressed in lung ECs (121).The genes encoding the S1P1 receptor or *S1P1*,the critical barrier-promoting receptor (122), and the inflammation-promoting S1P3 receptor, *S1P3*, a potential ARDS biomarker (77), exhibit several SNPs overrepresented in Blacks with severe asthma and ARDS (123). The S1PR1 SNPs alter EC responses to ARDS-relevant growth factors. The two *S1PR3* promoter SNPs reduce S1PR3 promoter activity, reduce plasma S1PR3 levels in sepsis and ARDS (78), and are associated with decreased risk for sepsis-associated ARDS (78).

Both candidate genes and GWAS identified SNPs in *ANGPT2* encoding ANGPT2 that are strongly linked to ARDS development (124, 125) and plasma ANGPT2 levels. Furthermore, in subjects of European ancestry with sepsis, *ANGPT2* variants predict plasma ANGPT2 levels coupled with ARDS risk (124, 125).

Finally, VEGF, also known as the vascular permeability factor, is a well-recognized permeability-inducing agonist in preclinical and clinical studies of ARDS. SNPs in *VEGFA*, encoding VEGF, and *FLT1*, encoding the tyrosine kinase receptor within the VEGF receptor family, are recognized as viable ARDS candidate genes (126, 127) and are associated with dysregulated plasma VEGF levels and higher ARDS mortality (127). Therefore, alterations in VEGF and FLT1 expression are mechanistically linked to the critical role of the endothelium in the pathophysiology of ARDS (127).

### 4.2. The Pulmonary Niche in Acute Lung Injury/Acute Respiratory Distress Syndrome: Single-Cell and Organ-Specific RNA-Seq

Single-cell analysis of the quiescent and activated alveolar endothelium reveals cellular complexity that is altered by inflammatory conditions such as ALI/ARDS. At least two previously indistinguishable capillary EC types have been defined: aerocytes and general capillary ECs (GenCap). Aerocytes are distinguished by a unique transcriptome, including the expression of the genes encoding the endothelin receptor (EDNRB), and the transcription factors T-box transcription factor 2 (TBX2) and forkhead box P2 (FOXP2) (9). Aerocytes are unique to the lung where they exhibit a large surface area that mirrors alveolar type 1 (AT1) cells in forming the blood–air barrier (128). In contrast to all other ECs, aerocytes do not express genes encoding major components of endothelial-specific Weibel-Palade bodies (vWF, SELP, EDN1), arguing against their central role in amplifying innate immune responses (9). In contrast, *GenCap* endothelium can be identified by the expression of genes related to lipid transport, innate immune responses, and cytokine receptors such as IL7R and IL18R1, implying a more active role in innate immune responses. Importantly, GenCap ECs are stem cells/progenitors for aerocytes, an essential function in ALI/ARDS repair (9, 128).

Capillary changes underlie ALI/ARDS pathogenesis, including hyperpermeability and coagulopathy, making it essential to understand how capillary cell heterogeneity in the quiescent endothelium, as well as dynamic changes in capillary cell composition as lung injury evolves/resolves, impacts disease pathogenesis. In chronic obstructive pulmonary disease (COPD), single-cell resolution identified the loss of terminal airway–enriched secretory cells and region-specific endothelial capillary cells combined with increased CD8+ T cells and increased IFN-γ signaling as the cellular basis of distal airway remodeling in COPD (129). Similarly, an improved understanding of the altered cellular architecture of the ALI/ARDS capillary could yield novel therapies to restore cellular organization. Additionally, relative expression of causally linked ALI/ARDS genes (see Section 4.1 for more on genetic influences) could mirror changes in specialized capillary cell abundance in evolving lung injury, informing timing and cellular targeting of future vascular therapies to limit off-target toxicities. This work is already being done in murine ALI models. A novel *Car4*-high EC has been described in regenerating alveolus regions and sites of maximal alveolar injury (130). C*ar4*-high ECs possess a unique transcriptome, including high expression of VEGF receptor genes, suggesting a role in angiogenesis related to alveolar regeneration.

### 4.3. Identifying High Vascular Injury Acute Respiratory Distress Syndrome: Insights from Neutropenic Acute Respiratory Distress Syndrome

A sobering fact for scientists hoping to therapeutically target the lung endothelium is that vascular biomarker and proteomic analyses reveal that a significant portion of ARDS subjects do not exhibit pathologic vascular responses and are unlikely to benefit from vascular-targeted therapies. Conversely, identifying high vascular injury-prone ARDS populations could (*a*) accelerate the discovery/approval of new vascular therapies, (*b*) allow for a reappraisal of vascular therapies that have previously failed, and (*c*) improve the unacceptably high mortality rates associated with high vascular injury ARDS.

Neutropenic ARDS, an ARDS population easily identified by an absolute neutrophil count of fewer than 1,500 cells/μL, fits this high vascular injury phenotype. With pathologic findings of diffuse alveolar damage and hyaline membranes, ARDS develops in subjects during prolonged neutropenia (131), and neutropenia is an ARDS risk factor (132). A unifying hypothesis is that aberrant inflammation and EC activation (see Section 2) drive neutropenic ARDS pathogenesis, validated in mice sequentially depleted of circulating neutrophils [anti-Ly6G monoclonal antibody (mAb)] and separately neutrophils/monocytes (anti-Gr1 mAb) that generate stepwise and paradoxical TNF-α hyperinflammatory responses to endotoxin (133). Similarly, neutropenic ARDS subjects, often pancytopenic in addition to neutropenic, demonstrate hyperinflammation, with 20-fold higher plasma IL-6 and elevated tumor necrosis factor receptor 1 levels compared to control ARDS subjects (132).

**Figure 4:**
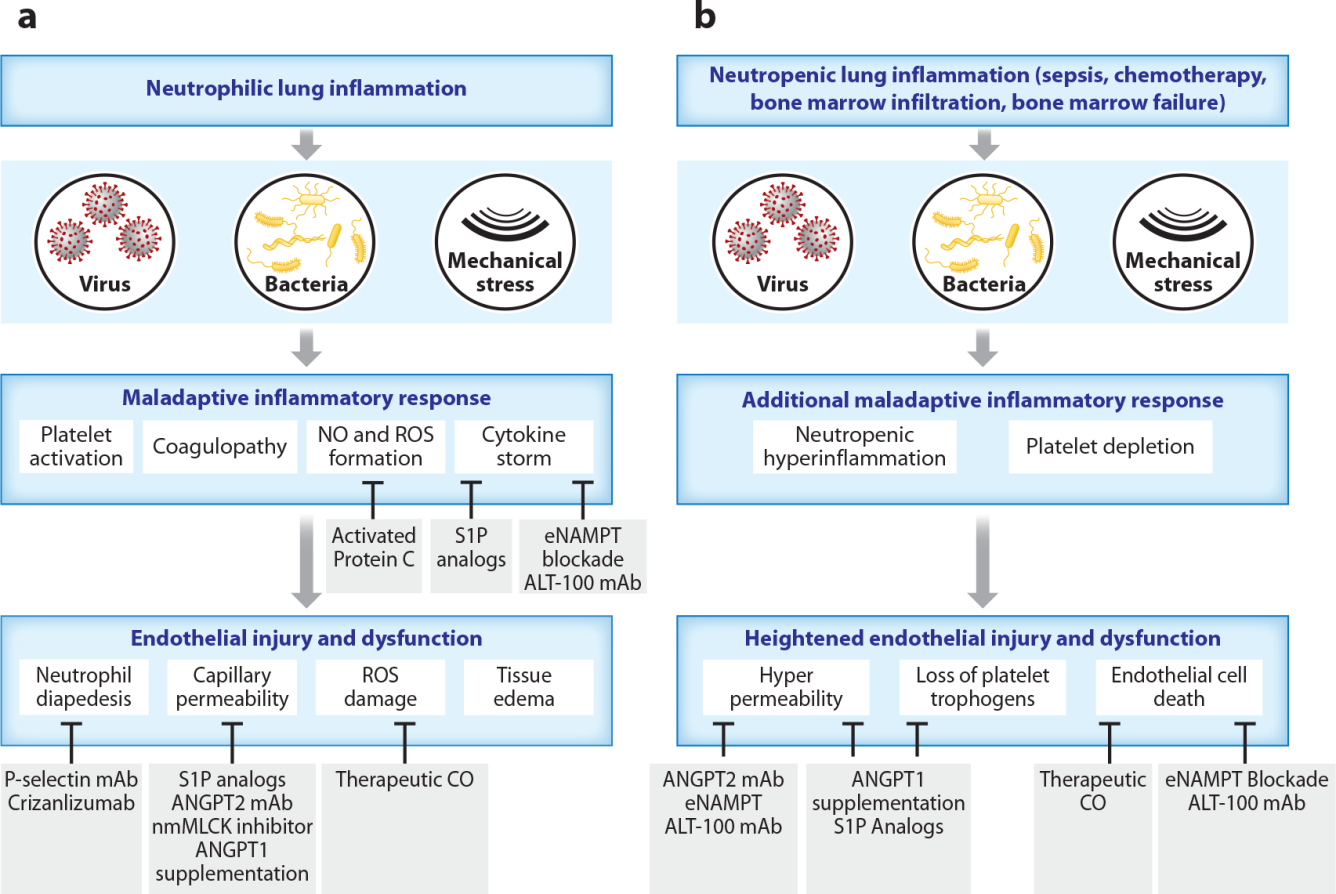
Schema for matching vascular-targeted therapies to ARDS patients stratified by neutrophil status. Prior large ARDS clinical trials designed to reduce ARDS severity and mortality have failed. (*a*) Shown are promising, albeit untested, vascular-targeted therapies with broad applicability to ARDS populations. (*b*) Matching vascular therapies to patients with vascular vulnerabilities, typified by neutropenic ARDS, may increase the likelihood of trial success, as would the incorporation of precision medicine approaches with biomarker- and genotype-based stratification. Abbreviations: ANGPT, angiopoietin; ARDS, acute respiratory distress syndrome; CO, carbon monoxide; eNAMPT, extracellular nicotinamide phosphoribosyltransferase; mAb, monoclonal antibody; nmMLCK, nonmuscle myosin light chain kinase; NO, nitric oxide; P-selectin, platelet-selectin; ROS, reactive oxygen species; S1P, sphingosine-1-phosphate.

In addition, loss of platelet-derived trophogens from the plasma (see Section 3), manifest in this population by high levels of thrombocytopenia, presents an additional vascular vulnerability in neutropenic ARDS. Important platelet trophogens, including plasma ANGPT1, PDGFA and PDGFB, and MMP9, are all dramatically lower, and the plasma ANGPT2/ANGPT1 ratio is dramatically higher in neutropenic ARDS plasma compared to ARDS controls (D.R. Price, unpublished data). These biomarkers identify impaired vascular barrier function and limited vascular repair as potential maladaptive leukocyte-independent mechanisms of vascular injury in neutropenic ARDS, resulting in worse outcomes (134). Vascular therapies that address hyperinflammation/hyperpermeability from exaggerated barrier disruptors or loss of platelet trophogens could be particularly beneficial in high vascular injury ARDS subjects typified by neutrophil- and platelet-depleted ARDS subjects (Figure 4).

## 5. LUNG ENDOTHELIUM AS A DRUGGABLE TARGET IN ACUTE RESPIRATORY DISTRESS SYNDROME: POTENTIAL VASCULAR-TARGETED THERAPIES

There are no ARDS pharmacotherapies approved by the US Food and Drug Administration (FDA), a grim realization dramatically highlighted in the global COVID-19 pandemic through multiple failed clinical trials. Sadly, ARDS care remains largely supportive, underscoring a serious unmet need for ARDS therapies that attenuate lung vascular permeability and inflammation. Despite this abysmal record of failure, current mechanistic advances indicate that lung EC barrier regulation/immune effector function may serve as attractive druggable targets to drive restoration of the integrity of the injured pulmonary circulation and to reduce ARDS mortality. For example, the nmMLCK isoform is an attractive ARDS/VILI therapeutic target, as nmMLCK inhibitory approaches [short interfering RNAs (siRNAs)] and nanoparticle delivery of peptide inhibitor of kinase (PIK), a nmMLCK kinase inhibitor, effectively reduce alveolar and vascular permeability and lung inflammation (45, 49, 135), suggesting the targeting of EC nmMLCK as a therapeutic strategy.

DAMPs are attractive therapeutic targets for attenuating ARDS severity/mortality given their significant influence on PRR-driven inflammation, including EC inflammation (detailed in Section 2). eNAMPT is a novel DAMP and highly druggable innate immunity inflammatory target that directly participates in ARDS/VILI pathobiology. eNAMPT plasma levels (29) and *NAMPT* SNPs (32, 118) are linked to human ARDS severity and mortality. Importantly, ALT-100, an eNAMPT-neutralizing humanized mAb currently in Phase 2a clinical trials for ARDS, was profoundly effective in reducing the severity of lung injury in murine, rat, and porcine ARDS/VILI studies, with profound attenuation of inflammatory cytokine production and increased lung permeability (136–138). Importantly, conditional EC-specific *NAMPT* knockout mice demonstrate that EC-derived eNAMPT is essential in evoking preclinical LPS/VILI lung injury (136).

In preclinical murine, rat, and canine ARDS/VILI studies, lung EC S1PR1 activation by intravenous-delivered S1P or S1P analogs rapidly initiates a signaling cascade that reorganizes the EC cytoskeleton, enhances junctional integrity, decreases alveolar edema formation, and markedly improves oxygenation (139). This finding was observed in other inflammatory models, including VILI (140, 141), ischemia/reperfusion (142), ionizing radiation (143), and traumatic brain death (144). Thus, S1P analogs are attractive and viable EC-focused molecular targets for preventing and ameliorating ARDS in critically ill patients in intensive care units.

PSGL1 encoded by *SELPLG*, a novel ARDS susceptibility gene identified in African Americans with coding SNPs, confers ARDS susceptibility (119) and is critically involved in PMN trafficking. PSGL1 is an attractive ARDS target with several approaches to PSGL1 neutralization that have all attenuated lung injury in preclinical ARDS/VILI models. These include the FDA-approved P-selectin-binding humanized mAb, crizanlizumab (119, 145), the human anti-PSGL1 mAb (CD162) (119), and TSGL-Ig, a novel recombinant tandem PSGL1 immunoglobulin fusion molecule (120).

The angiopoietin axis barrier-stabilizing protein ANGPT1 and barrier-destabilizing protein ANGPT2 are additional attractive targets for novel vascular therapeutics. Pathogenic depletion of ANGPT1 has been linked to ARDS outcomes, and therapeutic supplementation of ANGPT1 with the drug vasculotide attenuates leak in preclinical models (146, 147). The ANGPT1 analog AV-001 (a newer version of vasculotide) has reached Phase 2a clinical study in hospitalized patients with COVID-19. Additionally, the ANGPT2-blocking mAB Ly3127804 was assessed in hospitalized COVID-19 pneumonia patients but failed to reduce the number of ventilator days over placebo and was terminated (148).

Carbon monoxide (CO) is an endogenously produced gaseous molecule with important signaling roles in cellular physiology. CO is mainly produced during heme degradation by heme oxygenase enzymes (HO-1, HO-2). Although widely known as a poisonous gas, preclinical studies support inhaled CO (iCO) as a potential novel therapeutic in ARDS, with a recent Phase 1 study demonstrating the feasibility and safety of iCO in mechanically ventilated ARDS patients (149). A current Phase 2 trial is underway.

## 6. SUMMARY AND CONCLUSION

Lung ECs are a key target cell in ARDS pathobiology, serving as direct innate immune effectors, dynamically responding to extracellular environmental changes in addition to pathogens by participating in innate and adaptive immune responses highly relevant to ALI/ARDS. The transformation of quiescent anti-inflammatory monolayers with high barrier integrity into activated proinflammatory endothelium occurs via cell surface expression of PRRs (ligated by PAMPs and EC-derived DAMPs) and cytokine receptors (including EC-secreted cytokines) and amplifies the immune response. Loss of lung EC homeostasis can lead to unchecked procoagulant, profibrotic, and proinflammatory mechanisms with immune cell influxes into the lung interstitium, especially when mechanical ventilator-induced cellular stress prolongs the activated proinflammatory EC phenotype and vascular injury.

ECs are also the major contributor to ARDS severity and mortality via lung and systemic inflammation-induced increases in vascular permeability and multi-organ failure. This unremitting permeability and vascular barrier restoration are governed by the dynamic contractile function of the RhoGTPas-influenced, nmMLCK-driven EC cytoskeleton and its linkage to cell–cell and cell–matrix connections and paracellular lamellipodia with gap closure. However, the EC cytoskeleton provides the vascular system with the plasticity to respond to changing physiologic/pathophysiologic needs. Driven by critical barrier-enhancing endogenous agonists such as S1P, ANGPT1, and HGF, cytoskeletal responses are orchestrated to restore the integrity of the lung vasculature via Rac GTPase-elicited increases in cortical actin and lamellipodial protrusions, which accomplish paracellular gap closure.

Finally, previous vascular-targeted trials in ARDS have failed (150, 151), perhaps in part due to the inclusion of ARDS subjects with minimal vascular injury. The era of vascular targeting was arguably launched with Xigris or activated protein C, the landmark drug in severe sepsis. Fortunately, despite the striking failure of Xigris in Phase 3 trials (152), drug development targeting the lung vasculature has achieved major advances (Figure 4). Whether small molecules as potential ARDS-modulating modalities, such as with the nmMLCK peptide inhibitor PIK or biologic entities such as the eNAMPT-neutralizing ALT-100 mAb, the integrin β4-neutralizing mAb, the ANGPT2-neutralizing mAb, or the PSGL1-neutralizing biologic strategies, there has been profound progress in the development of ARDS relevant EC-targeted therapies. As ARDS mortality is unlikely to be significantly reduced by a single therapeutic modality, a mandate exists for the evaluation of potential therapeutic synergies between mechanistically derived lung EC barrier-enhancing approaches coupled to other ROS-, pyroptosis-, necroptosis- and inflammation-reducing strategies that collectively serve to restore the integrity of injured lung endothelium.

## Figures and Tables

**Table 1:** Single nucleotide polymorphisms (SNPs) identified in genes altering vascular inflammatory and permeability-regulatory pathways and associated with risk of acute respiratory distress syndrome (ARDS)

Gene	Name	Function	Race phenotypes	SNP site	ARDS risk	SNP function	Reference (s)
*MYLK*	Myosin light chain kinase	Endothelial/epithelial barrier regulation Angiogenesis Leukocyte trafficking Innate immunity Inflammation	AA	hcvl602689 MYLK_03 7 rs28497577 rs9840993 rs3796164 rs4678047 rs2700408	Increased	Increases nonmuscle *MYLK* promoter activity (rs2700408; rs11714297) Alters EC gap closure dynamics delaying lung EC barrier recovery (rs9840993) Alters secondary mRNA structure (rs9840993)	7, 46, 58, 108, 110, 111
			AA/EA	rs820336			
			EA	rs11718105 rs11714297 rs4678062			
*CTTN*	Cortaetin	Barrier maintenance	AA/AD	rs56162978	Increased	Reduced phosphorylation and delayed barrier recovery	47, 109
*NAMPT*	Nicotinamide phosphoribosyltransferase	NAD synthesis DAMP inflammation	EA	rs9770242	Increased	Unknown	32, 116–118, 154
			EA/SP	rs59744560 rs7789066	Increased	Increased promoter activity	
			EA/AA	rs61330082	Reduced	Reduced promoter activity	
*SELPLG*	Selectin P ligand	Leukocyte trafficking	EA/AA	rs2228315	Increased	Unknown	119, 120
*S1P3*	Sphingosine-1-phosphate receptor 3	Inflammation Barrier disruption	EA/ED	rs7022797 rs11137480	Reduced	Reduced promoter activity	122
*ANGPT2*	Angiopoietin 2	Barrier disruption Inflammation	EA	rs2515475	Increased	Unknown	124, 125
			EA/AA	rs1868554 rs2442598			
*VEGFA*	Vascular endothelial growth factor A	Barrier disruption Angiogenesis	EA	rs3025039	Increased	Decreased plasma *VEGF* expression	126
*FLT1*	Fms-related receptor tyrosine kinase 1	Barrier disruption Angiogenesis	SP/ED	rs9513106	Reduced	Unknown	127

Abbreviations: AA, African American; AD, African descent; DAMP, damage-associated molecular pattern; EA, European American; EC, endothelial cell; ED, European descent; NAD, nicotinamide adenine dinucleotide; SP, Spanish.
